# Correlation between Serum Uric Acid Level and Microalbuminuria in Type-2 Diabetic Nephropathy

**DOI:** 10.12669/pjms.336.13224

**Published:** 2017

**Authors:** Hina Latif, Adil Iqbal, Rabia Rathore, Nasir Farooq Butt

**Affiliations:** 1 Hina Latif, FCPS. Department of Medicine, Mayo Hospital, Lahore, Pakistan; 2 Adil Iqbal, FCPS. Department of Medicine, Mayo Hospital, Lahore, Pakistan; 3 Rabia Rathore, FCPS. Department of Medicine, Mayo Hospital, Lahore, Pakistan; 4 Nasir Farooq But, FCPS. Department of Medicine, Mayo Hospital, Lahore, Pakistan

**Keywords:** Diabetic nephropathy, Serum uric acid and microalbuminuria, Type-2 diabetes mellitus

## Abstract

**Objective:**

To measure the correlation between microalbuminuria and serum uric acid level in Type-2 diabetic nephropathy.

**Methods:**

This cross-sectional study was done in department of Medicine, Mayo hospital Lahore from August 2014 to February 2015. A total of 200 patients with Type-2 diabetic nephropathy were enrolled in the study. Demographic data and contact details were obtained. Serum Uric acid and microalbuminuria by albumin to creatinine ratio (ACR) in random urine sample was measured at the time of inclusion of patients. All the information was collected through a pre-defined proforma. Pearson correlation coefficient and t-test were used to assess correlation and significance respectively.

**Results:**

Out of 200 cases, 29%(n=58) were between 16-40 years of age while 71%(n=142) were between 41-65 years of age, Mean ± SD was calculated as 48.1±10.26 years, 48.5%(n=97) were male and 51.5%(n=103) were females, Mean serum uric acid level was calculated as 6.99±1.01 mg/dL while microalbuminuria was calculated as 5.63±1.08 mg/mmol, r value was 0.0838 which is a positive correlation.

**Conclusion:**

The results of our study concluded that level of serum uric acid and microalbuminuria are significantly correlated to nephropathy in patients having Type-2 diabetes mellitus.

## INTRODUCTION

Diabetes mellitus (DM) is one of the most prevalent health issues worldwide. Its prevalence is increasing, with more than 180 million people worldwide and it is supposed that it would be prevalent in 366 million people by the year 2030.^1^ Type-2 diabetes mellitus (DM) is correlated with a higher threat of cardiovascular disorders and atherosclerotic load.^2^ In Pakistan, the cumulative prevalence of Type-2 diabetes mellitus is 13.14%.^3^

Uric acid is produced by the enzymatic activity of xanthine oxidase and is the final product of purine metabolism.^4^ Xanthine oxidase produces oxidants in this process that may have a role in cardiovascular disease and kidney dysfunction.^5^ Approximately one-third of uric acid is degraded in the gut, and two-thirds is excreted by the kidneys.^6^ During uric acid production, oxygen free radicals are generated and therefore, uric acid may be a simple and useful clinical indicator of excess oxidative stress.^7^ Albuminuria is defined as the presence of a surplus quantity of serum proteins in the urine. This excessive amount of protein usually makes the urine frothy.^8^ Albuminuria is an indicator of renal injury. The existence of excessive amount of serum proteins in urine exhibits the inadequacy of reabsorption or impaired filtration by the kidneys. This condition, if prolong, may impair nephrons and may lead to development of albuminuria in diabetics.^9^

Diabetic nephropathy (DN) is still the most prevailing reason of end-stage renal disease (ESRD). There are numerous factors that are associated with the advancement of nephropathy in patients having Type-2 diabetes (T2D) including age, poor glycemic control, hypertension, and smoking.^10^ Of them, inflammation and endothelial dysfunction seems to have a basic contributing factor in the progression of DN.^11^ Recent observations suggest that uric acid is an element which may lead to inflammation and may play a pivotal part in endothelial dysfunction and results in the development of DN. The high mobility group box chromosomal protein 1 (HMGB1) expression and extracellular release in endothelial cells is caused by a rise in uric acid levels. The HMGB1 leads to endothelial dysfunction; it is an inflammatory cytokine which, when binds with the receptor for advanced glycation end-products (RAGE), induces an oxidative stress and inflammatory response, which leads to endothelial dysfunction.^12^

Zoppini and colleagues reported that in patients having Type-2 diabetes with conserved renal function, hyperuricemia seems to be an individual risk factor for the development of incident chronic kidney disease (CKD). Unlike patients without hyperuricemia, the study found that CKD (in terms of overt proteinuria) was extremely higher in patients having hyperuricemia (29.5 vs. 11.4%, p-value < 0.001).^13^

The aim of our study was to see the correlation of uric acid with microalbuminuria in Type-2 diabetes mellitus. A number of studies has been done globally however only few studies are available locally in Pakistan. A similar study was performed earlier in the province of Khyber Pakhtunkhwa, Pakistan but the sample size taken was quite small to check the relationship between albuminuria and uric acid in diabetic patients.^14^ On the other hand, our study was conducted in one of the largest public-sector hospital of the most densely populated province of Punjab, Pakistan. This also makes our study unique since there is also genetic diversity between the people residing in both provinces.

Early investigations and management of hyperuricemia in patients with diabetes may prevent their progression to overt nephropathy.

## METHODS

This cross-sectional study was conducted at department of Medicine, Mayo hospital Lahore from August 2014 to February 2015. The sample size of 200 cases was estimated using Type-I error as 5 percent and Type-II error as 10 percent taking an expected association between uric acid and microalbuminuria as 0.47. Non-probability purposive sampling technique was used. All patients aged 16-65 years of either gender and with Type-2 diabetic nephropathy [having microalbuminuria i.e. albumin to creatinine ratio (ACR) between 2 to 20 mg/mmol (in males) and 2.8 to 28 mg/mmol (in females)] were included in the study.

Patients with myocardial infarction (within past 3 months), impaired function of kidneys (serum creatinine level >1.5mg/dL or having macroalbuminuria i.e. for females ACR > 28 mg/mmol and in males ACR > 20 mg/mmol, severe valvular heart disease, heart failure, hypothyroidism, malignancy, alcoholism, gout or other inflammatory diseases, urinary tract infection, pregnant or menstruating females, history of fever or severe trauma within last 7 days and those using corticosteroid or cytotoxic drugs were excluded.

After attaining ethical approval from Institutional Review Board, King Edward Medical University, 200 patients fulfilling inclusion criteria were enrolled in the study. An informed consent was obtained and the objective of study, methods and outcomes were explained to each patient or attendant. Demographic data, contact details were obtained. For the measurement of serum creatinine, serum albumin and uric acid, venous blood samples were collected.

Microalbuminuria was measured by albumin to creatinine ratio (ACR) in random urine sample. The normal Albumin to Creatinine ratio (ACR) in healthy adult females is less than 2.8 mg albumin/mmol creatinine while it is less than 2.0 mg/mmol in healthy adult males. ACR ranging between 2 to 20 (in males) and 2.8 to 28 (in females) mg/mmol depicts microalbuminuria. Albumin to Creatinine ratio (ACR) levels which are above 28.0 mg/mmol in females and 20.0 mg/mmol in males correlates that an excess of 300 mg/day albumin level is present in urine (macroalbuminuria) and is consistent with overt diabetic nephropathy.

All the data was entered and analyzed using SPSS version 20. Continuous data like age of patient, serum uric acid level and microalbuminuria was expressed as Mean ± Standard Deviation. Frequency and percentages were calculated for gender. Correlation was calculated between serum uric acid level and albuminuria by pearson correlation. Data was stratified for duration of DM, age and gender. Post stratification t-test was applied to see the significance difference. P-value less than or equal to 0.05 was considered to be statistically significant.

## RESULTS

Age distribution of the patients was done which showed that 29% (n=58) were between 16-40 years of age while 71% (n=142) were between 41-65 years of age, Mean ± SD was calculated as 48.1±10.26 years. Patients were distributed according to gender, 48.5 % (n=97) were male and 51.5% (n=103) were females. Duration of diabetes mellitus was recorded as 58% (n=116) with ≤5 years of duration while 42% (n=84) had >5 years of duration of diabetes mellitus.

The results of different variables in patients of Type-2 Diabetic Nephropathy are shown in Table-I. Correlation between level of microalbuminuria and serum uric acid level in Type-2 diabetic nephropathy was determined. Mean serum uric acid level was calculated as 6.99±1.01 (mg/dL) while microalbuminuria was calculated as 5.63±1.08 (mg/mmol), r value was 0.0838 showing a positive correlation. P-value was calculated as 0.0001 (Table-II).

**Table-I:** The results of Variables in Type 2 Diabetic Nephropathic Patients.

*Variables*	*Minimum*	*Maximum*	*Mean ± SD*
Age (years)	32	65	48.1+10.26
Serum Creatinine (mg/dL)	0.6	1.4	0.8431± 0.191
Height (cm)	160	187	173.96 ± 7.261
Weight (kg)	52	88	69.35 ± 8.161
GFR (mL/min/1.73 m^2^)	61	118	98.150 ± 11.068
BMI (Kg/m^2^)	17.2	30.06	22.866 ± 2.0197
HbA1c (%)	6.5	8.9	7.619 ± 0.4774
Systolic BP (mm Hg)	105	150	128.99± 10.665
Diastolic BP (mm Hg)	50	100	74.48 ± 9.0

**Table-II:** Correlation between serum Uric Acid level and Microalbuminuria in Type-2 Diabetic Nephropathy (N=200).

	*Mean*	*SD*
Serum uric acid (mg/dL)	6.99	1.01
Microalbuminuria (mg/mmol)	5.63	1.08

r-value=0.0838, p-value: 0.0001

Stratification for age, gender and duration of disease was done. In patients aged 16-40 years, mean uric acid level and microalbuminuria were 6.95±1.02 (mg/dL) and 5.67±1.11 (mg/mmol) respectively (r value= 0.060) while in patients aged 41-65 years values were 7.05±0.89 (mg/dL) and 5.57±1.09 respectively (r-value= 0.060).

In male patients mean uric acid level and microalbuminuria were 6.92±1.12 (mg/dL) and 5.69±1.06 (mg/mmol) respectively (r-value= 0.143) while in females values were 7.05±0.89 (mg/dL) and 5.57±1.09 respectively (r-value= 0.025).

In patients having diabetes of less than 5 years duration, mean uric acid level and microalbuminuria were 7.07±0.98 (mg/dL) and 5.66±1.07 (mg/mmol) respectively (r-value= 0.164) while in patients with diabetes more than 5 years duration these values were 6.87+1.05 (mg/dL) and 5.58±1.09 respectively (r-value= 0.060) Table-III.

**Table-III:** Stratification for Age, Gender and Duration of Diabetes Mellitus (n=200)

		*Serum Uric Acid (mg/dL)*	*Microalbuminuria (mg/mmol)*	*r-value*
Age (years)	16-40	6.95±1.02	5.67±1.11	0.060
	41-65	7.05±0.89	5.57±1.09	0.060
Gender	Male	6.92±1.12	5.69±1.06	0.143
	Female	7.05±0.89	5.57±1.09	0.025
Duration of diabetes (years)	≤5	7.07±0.98	5.66±1.07	0.164
	>5	6.87+1.05	5.58±1.09	0.060

## DISCUSSION

Despite the recent advancement in the management of diabetes, nephropathy remains the most common cause of end stage renal disease (ESRD). Inflammation and endothelial dysfunction play an important role in the onset and advancement of DN. Recently serum uric acid is considered to be an inflammatory factor which may play a significant role in endothelial dysfunction.^11^,^12^ These findings have led us to find out the role of uric acid in the onset and progression of diabetic nephropathy.

We evaluated correlation between microalbuminuria and uric acid concentration in Type-2 diabetics. In our study 200 Type-2 diabetics were included. Mean serum uric acid level was calculated as 6.99±1.01 mg/dL while microalbuminuria was calculated as 5.63±1.08 mg/mmol, r-value was 0.0838 which shows a positive correlation.

Behradmanesh et al., established in a study that serum uric acid had an important affirmative correlation with diabetic nephropathy (Mean ± SE and median of proteinuria was 388 ± 28.7 mg/day and 303.5 mg/day respectively, while Mean ± SE of serum uric acid level was found to be 4.5 ± 0.15 mg/dL) in Type-2 diabetic patients. They concluded that in Type-2 diabetic patient’s uric acid level in serum plays a momentous role in causing nephropathy.^15^

Study done by Sunita Neupane and colleagues showed that serum uric acid concentration corresponds conclusively with Urinary Albumin Excretion (UAE) with an r-value of 0.323, and P-value less than 0.05. Positive correlation was also found with age (r-value= 0.337, p-value < 0.05), age at onset (r-value *=* 0.341, p-value < 0.05) and total duration of diabetes (r-value=0.312, p-value < 0.05). Multiple regression analysis was done which showed that serum uric acid concentration, systolic blood pressure, HbA1c and total duration of DM were independent determinants of UAE.^16^ Another study done by Suryawanshi and associates showed a positive association between urine microalbumin and levels of uric acid in serum (p<0.001), hence concluded that the levels of uric acid in serum and urine microalbumin were early diagnostic markers for kidney and atherogenic cardiovascular diseases, besides this, it was also very helpful in defining the prognostic monitoring of the disease in Type-2 diabetics.^17^

Hyperuricemia is also prevalent in patients with chronic renal failure. Indeed, various studies have shown that hyperuricemia may have a pathogenic role in the development and progression of chronic renal failure, rather than simply exhibiting decreased uric acid excretion from kidneys.^18^ In diabetic patients, serum uric acid early in the course of diabetes is significantly correlated with later development of persistent macroalbuminuria.^19^ This was also shown in our study that in patients having diabetes of less than 5 years duration, mean uric acid level and microalbuminuria were 7.07±0.98 (mg/dL) and 5.66±1.07 (mg/mmol) respectively (r-value=0.164) while in patients with duration of diabetes more than 5 years values were 6.87+1.05 (mg/dL) and 5.58±1.09 respectively (r-value= 0.060).

In the above discussion, positive correlation is found between microalbuminuria and levels of serum uric acid of Type-2 diabetic patients having nephropathy in our population which is in accordance with the results of other studies. Dr. Beena Unnikrishnan also showed that serum uric acid is a major pathological factor in the development of nephropathy in patients having Type-2 diabetes mellitus. In her study, out of the 50-study participant’s majority were females (60%). Mean ± SE of serum creatinine was 0.88 ± 0.038 mg/dL, Mean ± SE of serum uric acid was 4±0.12 mg/dl, and Mean ± SE of proteinuria was 382±24.7 mg/day (median=300.5 mg/day).^20^ Hyperuricemia is associated with kidney damage manifested by glomerular hypertrophy and sclerosis. We should develop some strategies to prevent possible renal failure in Type-2 diabetics. Therefore, diagnosing and treating hyperuricemia early in patients with diabetes may prevent their progression to renal failure. Uric acid levels can be decreased by using allopurinol, a xanthine oxidase inhibitor. Allopurinol inhibits the conversion of hypoxanthine to xanthine and finally to uric acid.^21^ At an average dose of 300 mg/day, allopurinol may lead to a decrease in levels of serum uric acid up to 30–40%, a decrement up to 60% can be achieved by administering the maximum dosage of 600 mg/day.^22^ This is shown in a study by David M. Maahs which showed that the use of allopurinol in lowering uric acid levels switch many of the detrimental effects of uric acid, this may lead to the suppression of Renin angiotensin aldosterone system, a reduction in oxidative stress, an enhanced Nitric oxide bioavailability, a better endothelial functioning, and a reduction in concentration of biomarkers for urinary inflammation.^23^ Similar results were seen in the research done by Bose B et al.^24^ The study depicted that Uric acid-lowering therapy in combination with allopurinol may retard progression of chronic kidney disease. Goicoechea M et al,^25^ did a study, in which they incorporated a total of 113 subjects, out of the total nearly 37% of patients were diabetic and they had a glomerular filtration rate of less than 60 ml a minute with a stable kidney functioning (in past 3 months, serum creatinine increment is <50%) relatively. The patients were then administered with 100 mg/day of allopurinol for 2 years. When the study concluded, an increased GFR was observed. On average, by 1 ml/min in the treatment arm versus a 3 ml/min loss in the placebo arm. Thus, treating hyperuricemia in Type-2 diabetics may help in preventing the progression to renal failure.

## CONCLUSION

We concluded that levels of serum uric acid and microalbuminuria are significantly correlated in nephropathy in Type-2 diabetic patients. Serum uric acid level can not only be used as early diagnostic marker but also for the prognostic monitoring of diabetic nephropathy. However, our findings are primary which needs some other trials to validate our results.

### Authors’ Contribution

**HL** conceived and designed the manuscript.

**AI and RR** did data collection and manuscript writing.

**NFB and RR** did statistical analysis & editing of manuscript.

**AI** did review and final approval of manuscript.
